# Supplementary material for: Food Parenting Practices and Feeding Styles and Their Relations with Weight Status in Children in Latin America and the Caribbean
Source: Int J Environ Res Public Health. 2022 Feb 11;19(4):2027. doi: 10.3390/ijerph19042027 (PMC8871894; doi:10.3390/ijerph19042027)
Supplement: Supplementary file 1 [file ijerph-19-02027-s001.zip › ijerph-1460168-supplementary.pdf]

Table S1. Data extraction of studies that met the selection criteria of the systematic review.

Author's (year) Country	Study Design & Country	Sample's Characteristics	Recruitment	Study Aim(s)	Measures of Feeding Practices and Feeding Styles	Measures of Child Weight Status	Main Findings
Study 1 (2019) [34]	Cross-sectional<br><br>Brazil	927 mothers/ children (50.9% boys)<br><br>Child age: 2-8-years; *M* = 4.98y (SD ± 1.8)<br><br>Mother age: 37.77y (SD ± 5)<br>Father age: 40.54y (SD ± 6.2)	Private schools São Paulo and Campinas, Brazil and from predominantly high SES and high education level	Examine relations between maternal use of pressure to eat and anthropometric, sociodemographic and behavioral characteristics	Pressure subscale (CPFQ) used for maternal self-report<br><br>CFPQ previously validated (adapted in Brazil)	BMI and BMIz calculated (WHO standards).	Negative association between pressure to eat and child BMIz. One unit increase of child BMI z related to a mean decrease of .86 likelihood of maternal use of pressure to eat (95% CI 0.77 to 0.96, p < .008).<br><br>One-point increase in maternal BMI, 0.93 mean decrease observed for maternal pressure (95% CI 0.89 to 0.97, p < .001).<br><br>Strongest factor associated with maternal pressure to eat was the child eating too little when mother was not around.
Study 2 (2018) [42]	Cross-sectional<br><br>Brazil	1071 parents/ children<br><br>984 mothers<br>87 fathers<br><br>410 (38.28%) parents of preschoolers	14 Brazilian private schools	Investigate associations of parent feeding behaviors, more distal familial influences (e.g., SES, maternal/pat	Brazilian CFPQ version; 6 subscales:<br>- Healthy eating guidance<br>- Monitoring<br>- Emotion regulation/food as reward<br>- Restriction for weight-control	Child BMI z-scores calculated (WHO standards)<br><br>Parental report of child's height, weight for anthropometric data	Preschoolers: negative associations between child BMIz and healthy eating guidance (β = -.36, p < .05) & pressure (β = -.22, p < .05).<br><br>School-age children: positive associations between child BMIz and

		661 (61.72%) parents of school-aged children<br><br>Child age:<br>Preschool: 2–5y, n = 397<br>school-aged: 6–8y, n = 618<br><br>Mother age: 36.36y<br>Father age: 38.81y<br><br>Parental education level & SES predominantly high		ernal weight) with child BMIz	- Restriction for health<br>- Pressure		restriction for health (β = .09, p < .05) and restriction for weight control (β = .36, p < .001)<br><br>Preschool- & school -age children: positive associations between child BMIz and restriction for weight control (β = .28, p < .001), restriction for health (β = .11, p < .05), and negative association between pressure (β = -.12, p < .05).
Study 3 (2014) [36]	Cross-sectional<br><br>Mexico (Northeast)	558 mothers/children<br><br>Child age: 3-11 years; *M* = 7.38y (SD ± 2.55)<br><br>Girls: 50.54% (n = 282)<br>Boys: 49.46% (n = 276)<br><br>Mother age: 34.37y (SD ± 6.90)<br>Mother education: 12.52y (SD ± 3.28)	2 preschools & 2 public schools in an urban area	1) Describe MEES<br>2) Determine relation between MEES and child nutritional status (BMI and body fat %)<br><br>3) Verify whether MEES differs according to child nutritional status	PEAS validated scale; evaluate frequency of feeding strategies (5-point Likert scale):<br>- Limit setting (limit amount of soda child drinks);<br>- Monitoring Control (pressuring child to eat);<br>- Reinforcement (praise child for eating healthy snacks)	BMI & body fat % used as indicators of child weight status.<br><br>BMI percentiles (WHO Growth standards)<br><br>Body fat % measured through bioelectric impedance.	Mothers exerted greater control (pressure to eat) over obese children and greater reinforcement (congratulating due to healthy eating) in underweight children.<br><br>Limiting, control and discipline associated with higher child BMI
Study 4 (2009) [40]	Cross-sectional<br><br>Chile	1029 mothers/children<br><br>Girls: 51%	Public nurseries with early	Assess association between childhood	Adapted CFQ version for maternal feeding practices (5-point Likert scale):	Mother & child heights & weights measured by trained assistants.	Direct relation between children's BMIz with restriction (rho = .19 in

		Boys: 49%<br><br>Child age: 3.1-5.7y; *M* = 4.3y (SD ± 0.3)<br><br>Mother age: 19–50y; *M* = 31.8y (SD ± 7.0)<br><br>Predominantly middle-low & low SES/vulnerable background	education services.	anthropometric measures with mothers' BMI and maternal feeding practices	- Restriction<br>- Pressure to eat<br>- Monitoring<br>- Concern for child weight<br>- Perceived responsibility	WHR<br><br>BMIz calculated (WHO Growth standards)	boys, and rho = .27 in girls, p < .001).<br><br>Negative relation between pressure to eat (rho = -.30 in boys, and rho = -.36 in girls, p < .001) and BMIz and WHR (rho = -.19 in boys, and rho = -.27 in girls, p < .001).<br><br>In girls and boys, high pressure to eat associated with a lower likelihood of the child to be obese.<br>Only in boys, restriction was related with higher likelihood of being obese.
Study 5 (2009) [37]	Cross-sectional<br><br>Chile	232 parents/ children randomly selected<br><br>Girls: 53.9% Boys: 46.1%<br><br>Child age:<br>Girls: M = 11.91y (SD ± 1.56)<br>Boys: M = 11.98y (SD ± 1.51)<br><br>Mothers from low SES	Urban schools	Evaluate maternal attitudes and child feeding practices in relation to child BMI.<br><br>Determine the long term effect of maternal attitudes and child feeding practices on children's weight status, assuming	7 subscales of CFQ, only 3 involved feeding practices:<br>- Restriction<br>- Pressure to eat<br>- Monitoring<br>Concern for child weight<br>- Perceived child weight	BMIz calculated (CDC reference)	In boys, pressure to eat negatively correlated with BMIz in cross-sectional analyses (r = -0.21, p < 0.05), and mothers of children with normal weight used more pressure to eat.<br><br>Restriction, pressure to eat and monitoring did not account for any variance in child BMIz in retrospective analysis.<br><br>A 37% (boys) and 45% variance (girls) in the

				that maternal attitudes and child-feeding practices persist over time.			change of BMIz between ages 9-12 explained by perceived child weight and concern for child weight.
Study 6 (2017) [35]	Cross-sectional<br><br>Mexico	566 mothers/ children<br><br>Child age: *M* = 4.47y (SD ± .79)<br><br>Mother age: *M* = 30.37y (SD ± 6.12)<br><br>Mother education average: 10.89y (SD ± 2.93)<br><br>Schools randomized according to preschool age<br><br>Children were selected through stratified randomized sampling according to size of stratum, institution and group size	Schools from two states of Mexico	Assess reliability of the Caregiver Feeding Style Questionnaire (CFSQ).<br><br>Verify association between MPCW and child' nutritional status.<br><br>Verify differences between MPCW and child's BMI.<br><br>Verify association between MPCW and MFS.	CFSQ; 19 items (Likert-type responses) grouped into 2 dimensions (demandingness and responsiveness).	Child BMI percentiles (WHO Growth standards)	[illegible]ose parents had [illegible] and indulgent feeding [illegible]e highest mean of BMI.<br><br>Misperception of child weight status in children with overweight/obesity found more frequently in mothers with an uninvolved feeding style
Study 7 (2019) [39]	Cross-sectional<br><br>Brazil	927 mothers/children<br><br>Girls: 49.08%<br>Boys: 50.92%	Registered in schools of urban cities.	Identify associations between maternal restrictive feeding practices for	Subscale maternal weight restriction from a previously adjusted CFPQ to be used in Brazil (self-report).	BMIz (WHO Growth standards)	Restriction exerted by mothers when child weight status was in the category of overweight/obesity/severe obesity (OR = 2.18, $p < 0.001$).

		Child age: 2-8y<br>≥ 5y: n = 542 (58.42%)<br>< 5y: n = 385 (41.53%)<br><br>Mother age:<br>> 38y: n = 414 (44.85%)<br>≤38y: n = 509 (55.15%)<br><br>Father age:<br>> 40y: n = 409 (45.75%)<br>≤40y: n = 485 (54.15%)<br><br>Parental education:<br>Mothers finished college: 88.5%<br>Fathers finished college: 81.5%<br><br>Families from high SES		child weight control and sociodemographic, behavioral, dietetic, and anthropometric characteristics.			Restrictive feeding practices for weight control were independently associated with mother perception of child being a little overweight/overweight obese (OR = 4.61, p = 0.001).
Study 8 (2011) [33]	Cross-sectional<br><br>Brazil	109 caregivers/children<br><br>Girls: 51.4%<br>Boys: 48.6%<br><br>Child age:<br>6-10y; *M* = 8.2y<br><br>Caregivers:<br>Mothers: 85.3%<br><br>Diverse SES	Schools	Study relationships between childhood excess weight and parental attitudes.	CFQ subscales:<br>- Pressure to eat<br>- Monitoring<br>- Restrictions	Child weight status (CDC reference)	Negative association between pressure and child excess weight.<br><br>Parents of children with excessive weight tended to apply more food restriction and monitoring to their children to eat than parents of children with a healthy weight.

		Caregiver education not reported					
Study 9 (2008) [38]	Case-control study<br><br>Brazil	100 caregivers/children<br><br>Child age:<br>6-10y; *M* = 7.8y<br><br>Child weight status:<br>Normal weight: n = 50<br>Obesity: n = 50<br><br>Mother education: not reported	Private (n = 7) and public (n = 18) urban schools	Identify risk factors for obesity in children.	Instrument (face-to-face interviews) created by the authors:<br>- Eating together as a family<br>- Constant limitation of food ingested by the child<br>- Having schedule to eat<br>(self-report)	Categorization of child weight based on BMI (cutoff points of CDC reference)	Parents' constant restriction on the amount of foods children consume associated with a higher risk for child obesity (OR: 62.9; p = .0012).<br><br>Mother overweight and frequent snack, and child consumption in commercial establishments associated with child obesity.
Study 10 (2019) [41]	Longitudinal study<br><br>Mexico	91 parent/children<br><br>Child age:<br>2–5y, *M* = 3.8y<br><br>Child sex:<br>Girls: 51.6%<br><br>Mothers: 98.1%<br>Fathers: 1.9%<br><br>Parent age:<br>*M* = 31.7y (SD ± 7.1)<br><br>Parents with high education level (12y): 46.2%	Public or private daycare centers and kindergartens	Evaluate association between FPPs and child weight status and the directionality of this association at 1-year follow-up	CFQ subscales:<br>- Monitoring<br>- Restriction<br>- Pressure to eat<br>- Parents' perceived responsibility<br>- Concern about child's risk for overweight<br>- Food used as a reward (from the CFQ used in Australian preschoolers)<br><br>TFQ:<br>- Indulgent feeding subscale	BMIz calculated (WHO standards)<br><br>WC measured and compared with the 90th %ile of the Third National Health and Nutrition Examination Survey of Mexican American children.	In boys, higher baseline BMI z was associated with more indulgent feeding (β = .23; p = .02) at follow-up.<br><br>In the whole sample (β = .36, p = .001) and in boys (β = .39; p = .008) higher indulgent feeding was associated with higher BMI after 1 year follow-up.<br><br>In girls, higher use of food as a reward at baseline positively associated with higher BMIz after 1 year follow-up (β =.31; p = .04). Food restriction at baseline predicted lower BMIz at follow-up (β = -.34; p = .03).

SES: Socio-economic Status; CFPQ: Comprehensive Feeding Practices Questionnaire; BMI: Body Mass Index; BMIz: z-scores of Body Mass Index; WHO: World Health Organization; CDC: Center for Control Disease; CFQ: Child Feeding Questionnaire, CFSQ: Caregiver´s Feeding Style Questionnaire; PEAS: Parental Strategies for Eating and Activity Scale, MPCW: Maternal perception of child weight status; MFS: Maternal feeding style; MEES: Maternal eating and physical activity strategies; TFQ: Toddler Feeding Questionnaire, FPPs: Food parenting practices; WC: Waist circumference; WHR: Waist-to-height ratio; OR: Odds ratio; SD: Standard deviation.
